# Machine Learning in Automated Monitoring of Metabolic Changes Accompanying the Differentiation of Adipose-Tissue-Derived Human Mesenchymal Stem Cells Employing ^1^H-^1^H TOCSY NMR

**DOI:** 10.3390/metabo13030352

**Published:** 2023-02-27

**Authors:** Lubaba Migdadi, Nour Sharar, Hanan Jafar, Ahmad Telfah, Roland Hergenröder, Christian Wöhler

**Affiliations:** 1 Image Analysis Group, TU Dortmund, 44227 Dortmund, Germany; 2 Leibniz-Institut für Analytische Wissenschaften—ISAS-e.V., 44139 Dortmund, Germany; 3 Cell Therapy Center, University of Jordan, Amman 11942, Jordan; 4 Department of Anatomy and Histology, College of Medicine, University of Jordan, Amman 11942, Jordan; 5 Nanotechnology Center, The University of Jordan, Amman 11942, Jordan

**Keywords:** machine learning, novelty detection, 2D NMR, mesenchymal stem cells (MSCs), adipogenic differentiation, osteogenic differentiation, metabolic dynamics, TOCSY, metabolomics, non-targeted metabolic profiling

## Abstract

The ability to monitor the dynamics of stem cell differentiation is a major goal for understanding biochemical evolution pathways. Automating the process of metabolic profiling using 2D NMR helps us to understand the various differentiation behaviors of stem cells, and therefore sheds light on the cellular pathways of development, and enhances our understanding of best practices for in vitro differentiation to guide cellular therapies. In this work, the dynamic evolution of adipose-tissue-derived human Mesenchymal stem cells (AT-derived hMSCs) after fourteen days of cultivation, adipocyte and osteocyte differentiation, was inspected based on ^1^H-^1^H TOCSY using machine learning. Multi-class classification in addition to the novelty detection of metabolites was established based on a control hMSC sample after four days’ cultivation and we successively detected the changes of metabolites in differentiated MSCs following a set of ^1^H-^1^H TOCSY experiments. The classifiers Kernel Null Foley-Sammon Transform and Kernel Density Estimation achieved a total classification error between 0% and 3.6% and false positive and false negative rates of 0%. This approach was successfully able to automatically reveal metabolic changes that accompanied MSC cellular evolution starting from their undifferentiated status to their prolonged cultivation and differentiation into adipocytes and osteocytes using machine learning supporting the research in the field of metabolic pathways of stem cell differentiation.

## 1. Introduction

Mesenchymal stem cells (MSCs) are multipotent stem cells with a high capacity to proliferate and differentiate, while exhibiting low immunogenicity and providing immunosuppressive properties [1]. These potentials put MSCs in the lead as a promising candidate for several innovative strategies of cellular therapy and tissue engineering. MSCs are obtained from several body tissue, and their potential in regeneration and differentiation is highly dependent on their [2,3]. Adipose tissue is considered a highly valued source to isolate MSCs being a byproduct that generates a good yield of primary cells, with high potential to proliferate and differentiate. Therefore, adipose tissue-derived MSCs are widely used in tissue engineering and regenerative medicine [4]. Metabolic adaptation of MSCs is highly dependent on their surrounding environment; MSCs cultivated under hypoxic conditions show limited proliferation rate and high production of glycolytic enzymes, while under normoxic conditions, they show high proliferation rate and an additional reliance on oxidation phosphorylation during glycolysis; described as Warburg effect [5]. Alternatively, studies have shown that the differentiation of MSCs into osteocytes is negatively affected by normoxic conditions [6]. The switch between the glycolytic and oxidative phosphorylation pathway shows the flexibility of MSCs in adapting a metabolism that enables them to fulfil regenerative and/or immunomodulatory roles at specific sites and environments. New approaches are required to reveal novel biomarkers and information in the metabolism of MSCs and to monitor the dynamics of their metabolism in response to stimuli, and metabolic adaptation associated with several biological processes, including differentiation [7,8]. This information may unveil their behavior which would enable researchers to control and guide these cells toward successful tailor-made therapies through providing the proper culture conditions and handling [9].

Nuclear magnetic resonance (NMR) spectroscopy is a powerful technique for the identification of the components of complex mixtures consist of small molecules, like metabolites in a mixture sample. NMR has proven its vital and powerful role as an analytical technique in metabolomics extracted from biofluid, tissue extract, or semisolid samples such as intact tissues or organs [10]. Chemical shifts are fingerprints that characterize the chemical composition of a biological compound [10,11]. The non-destructiveness and the reproducibility of NMR results enables high-throughput identification and quantitative accuracy of the metabolic concentration in biological mixtures [10,12,13]. However, due to the low sensitivity of NMR, spectral resolution and spectral overlapping, obtaining the metabolic profiling data from the NMR spectra is one of the main challenges in analyzing complex biological mixtures. Overlapping of the NMR signal and the shift in the NMR peaks are affected by the pH and ionic strength variations of the biological sample in which the metabolites are measured [13,14,15]. Two-dimensional NMR (2D NMR) has a significant resolving ability through adding a second frequency domain and dispersing the peaks into this added dimension [10]. Nevertheless, metabolic profiling of a 2D NMR spectrum with low concentration or overlapped peaks is an elaborate task [16]. Moreover, the analysis of biological sample is related to the complexity of biological mixtures, phase and baseline distortion and noise [15,17]. The 2D NMR TOCSY (Total Correlation Spectroscopy) experiment provides correlations between all the protons in the spin system allowing distinguish spin systems from different molecules [10]. Still, due to the dense content of 2D NMR spectrum of complex mixtures, manual analysis is a highly demanding task and it is dependent on the researcher’s experience [18].

In this work, machine learning has been applied to automate the monitoring of the MSCs differentiation and to resolve the convolution of the associated ^1^H-^1^H TOCSY NMR spectra. The analysis is based on observing the accompanying differentiation of AT-derived hMSCs cultivated in MSCs basal culture media in addition to their adipogenic or osteogenic differentiation. Identification of compounds related to MSCs differentiation based on non-targeted metabolic profiling is a significant task and has the potential to enable effective stem cell therapy [7]. Non-targeted metabolic profiling is an all-inclusive and comprehensive analysis of the whole NMR spectrum which requires the intensive analysis of 2D NMR TOCSY profiles to reveal novel occurrences of metabolites in response to various conditions and stimuli [19,20]. Methodologically, an unbiased classification approach is mandatory to overcome variations in the biological mixtures and the corresponding complexity of the NMR-generated data. Introducing machine learning as an analysis tool for 2D NMR appears to be a reasonable approach.

Several computer implementations have been proposed to enable NMR spectral processing and cross-peak identification of 2D NMR spectra. The COLMARm [21] web server is an online available platform that incorporates three types of 2D NMR spectra for the purpose of simultaneous analysis. COLMARm operates in two stages. First, an HSQC (Heteronuclear Single Quantum Coherence) spectrum is uploaded by the user, being compared against a unified database from the Biological Magnetic Resonance Data Bank (BMRB) [22] and the Human Metabolome Database (HMAB) [11] and a matched list of metabolites is created. In the next step, the matched list is validated against the corresponding TOCSY and/or HSQC-TOCSY spectrum. A Bayesian framework is used for the problem of the assignment of peaks in 2D NMR spectra. In [23], 2D NMR spectrum were modeled as a mixture of bivariate Gaussian densities. To estimate the positions of the peaks, the adaptive Markov chain Monte Carlo (MCMC) algorithm was used. A list of candidate peaks of the highest amplitude was created and the posterior probability of each candidate peak was calculated [23]. Another peak assignment approach which incorporates the shape of the peak on the 2D spectrum was introduced in [24], where images of the peak were translated into a matrix of features through shape mapping. These features are trained and tested using a SVM classifier [24]. Neural networks have utilized in NMR for the reconstruction and denoising of spectra, chemical shift prediction and automatic peak picking [25]. Mostly, these applications are implemented using mainstream libraries, such as TensorFlow [26,27,28] or the MATLAB Deep Learning Toolbox [29]. For the purpose of chemical shift prediction, multiple types of features have been used as a feature space for the training dataset. SMART and SMART 2.0 [30,31] are based on training a deep convolutional neural network (CNN) of Siamese architecture [32] to characterize new compounds, in addition to annotate known compounds in biological mixtures. Another tool that uses CNN to analyze 2D NMR is NMRNet [33]. NMRNet starts by sorting the cross peaks according to their intensity and eliminating peaks with low intensities. The images of the selected peaks are fed as cropped segments from the spectrum to a CNN. The output of the CNN is a sigmoid function that indicates the probability of the peak assignment [33]. [34]. Stem cell osteogenic differentiation of hMSCs for 21 days based on 1D NMR has recently been studied [7,35]. They mainly considered the lipidomic and amino acid characterization of osteogenic stem cells using Principal Component Analysis (PCA) and partial least squares discriminant analysis. Human embryonic stem cells were studied to monitor the intracellular and extracellular metabolic dynamics through directed and non-directed differentiation using 1D NMR. Similarly, PCA, least squares analysis and the ANOVA test were used to compare the differentiated and undifferentiated cells [36,37].

## 2. Materials and Methods

### 2.1. Machine Learning and Novelty Detection

In this work, the procedure creates an automatic metabolic profiling system of 2D NMR TOCSY spectra based on machine learning methodologies. The Kernel Null Foley–Sammon Transform classifier (KNFST) and Kernel density estimation (KDE) were tested to monitor the dynamic evolution of adipose-tissue-derived human MSCs. Novelty Detection (ND) or outlier detection is the task of distinguishing new samples that differ from the data on which the classifier has been trained. ND is involved in applications where new categories or classes are expected to appear in the future. Although the practicable training dataset is complete and contains all classification information at a given time, it is inapplicable to encompass all variations of the possible classes that might be encountered. Therefore, the classifier is supposed to detect new extreme conditions rather than classifying them into already-available classes. In the former situation, the training model developed during the training phase is not representative of the actual classification problem and the domain of expected categories. Extreme situations could be the emergence of unknown or unexpected metabolites during the dynamic biological evolution of samples, fault detection in industrial systems or the detection of new words in hand-writing applications [38,39,40]. A vital principle in ND is the novelty threshold which acts as a discrimination criterion between recognized and novel categories. The novelty threshold defines a decision function that is applied on the output scores of the classifiers [38,40,41].

The KNFST classifier is based on achieving the best separability between classes by maximizing the between-class scatter $S_{b}$ and minimizing the within-class scatter $S_{w}$ in a high-dimensional space φ. The objective of KNFST is to learn a discriminate projection direction ω which is calculated under the conditions $\omega^{T}S_{w}^{\varphi }\omega =0$ and ${\;\omega }^{T}S_{b}^{\varphi }\omega >0$. The previous conditions assure the best discrimination between classes [42,43,44,45]. Class identification can be achieved by calculating the Euclidean distance between the center of the projected training instances and the projection of the tested sample. KDE is a non-parametric probability-based classifier which measures the density of independent and identically distributed random points within a neighborhood range defined by the neighborhood width h. The Parzen window estimator [46] is a common KDE approach in which the class-conditional probability p(x) is calculated as a linear combination of the neighboring kernels k at each point in the dataset x. Parzen estimators can be defined as $p\left(x\right)=\frac{1}{N}\sum_{i=1}^{N}\frac{1}{h^{d}}k\left(\frac{x-x_{i}}{h}\right)$, where N and d define the number of samples x in the training data and the dimension of the feature space, respectively. The bandwidth h defines the smoothness of the kernel function known as the Parzen window [47]. KDE and KNFST have previously been used in the metabolic profiling of 2D TOCSY spectra of breast cancer tissue samples [45,48].

### 2.2. Sample Preparation and Experimentation

AT-derived hMSCs were obtained from the Cell Therapy Center (CTC)/The University of Jordan. The samples belong to consenting healthy females in the age range of 35–43, and the donors’ recruitment and sample collection were approved by the Institutional Review Board, the University of Jordan (IRB: CTC/1-2020/04 and approved on 10 March 2020).

#### 2.2.1. Cultivation of AT-Derived hMSCs

MSCs were maintained in basal MSCs culture media composed of alpha MEM medium with Earle’s Salts (Gibco) supplemented with 5% human platelet lysate (hPL), at a concentration of 3 I.U Heparin-Sodium 5000 I.U/mL, 1% penicillin streptomycin and 2 mM L-glutamine [49]. The cells were cultured in an adherent plate at a seeding density of 4000 cells/cm^2^, and subculture was performed every time the cells reached a confluence of 80% until reaching cell division in passage number 4 (P4). The passage number indicates the number of times that cells have been collected and recultured into new cell culture flasks [50].

#### 2.2.2. Adipogenic and Osteogenic Differentiation of AT-Derived hMSCs

**Figure 1:**
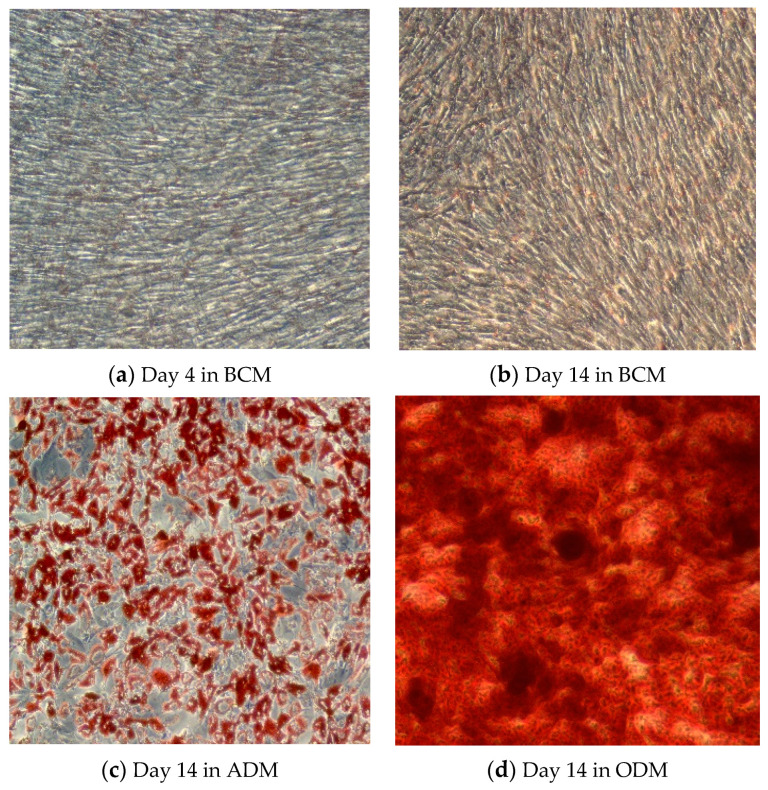
Light microscopy images (10x magnification, using inverted microscope, Zeiss, Oberkochen, Germany) of (**a**) AT-derived hMSCs after 4 days, and (**b**) 14 days of cultivation in basal culture media (BCM). (**c**) Oil red staining illustrating adipogenic differentiation of AT-derived hMSCs after 14 days of cultivation in adipogenic differentiation media (ADM). (**d**) Alizarin red staining illustrating osteogenic differentiation of AT-derived hMSCs after 14 days of cultivation in osteogenic differentiation media (ODM).

AT-MSCs were induced to differentiate into adipocytes or osteocytes using StemPro Adipogenesis and the Osteogenesis Differentiation Kit (Gibco), respectively, as described by the manufacturer. In brief, MSCs at P4 were cultivated in MSCs basal culture media (BCM) at a seeding density of 4000 cells/cm^2^. When cells reached 70% confluence, basal culture media was aspirated and the cells were washed twice with PBS, before the addition of complete adipogenic (ADM) or osteogenic differentiation media (ODM). The cells were maintained in standard culture conditions (37 °C, 5% CO_2_) in a humidified incubator for 14 days, while refeeding the cells every 3–4 days with completely fresh media. Through the duration of differentiation, morphological changes in MSCs were monitored using inverted microscopy. To confirm the differentiation of MSCs into adipocytes and osteocytes at the end of the differentiation duration, the generated monolayer of adipogenic- or osteogenic-induced MSCs went through a staining procedure using oil red O for adipocytes, or Alizarin red staining for osteocytes [51]. Oil red staining illustrates the internal neutral lipids generated in adipocytes [52,53], whereas alizarin red staining illustrates mineral deposits, such as calcium, generated by osteocytes [54]. BCM is supposed to maintain the stemness of MSCs without triggering their differentiation, and this was confirmed by the lack of coloration in AT-derived MSCs after 4 days of cultivation, as seen in Figure 1a. However, prolonged culture duration of AT-derived MSCs triggered their differentiation even in BMC which is detectable through the formation of lipid droplets and a faded oil red staining on Figure 1b. As seen in Figure 1c, MSCs cultivated in ADM for 14 days showed a clear alteration in their morphology due to the formation of large oil droplets in their cytoplasm as presented by the intense oil red staining. On the other hand, MSCs cultivated in ODM exhibited an intense deposition of minerals represented by the intense alizarin red staining, as shown on Figure 1d.

#### 2.2.3. Intracellular Metabolites Extraction

Following differentiation, intracellular metabolites were extracted using the methanol extraction method, as previously described [55]. Briefly, differentiation media were aspirated, and the cultured cells were washed three times with phosphate-buffered saline (PBS). Immediately after washing, absolute methanol stored at −20 °C and water ice were added to the cells in a ratio of 2 parts:0.8 parts MeOH:H_2_O to quench metabolism. Culture plates were stored at −80 °C for 10 min, then, the cells were scraped off the cell culture plate, and the obtained cells/methanol mixture were centrifuged at a speed of 14,000 rpm for 10 min. To obtain the intracellular metabolite in powder form, the samples were lyophilized, and the obtained powder from each sample was stored at −80 °C until further use [51].

### 2.3. High Resolution 1D and 2D NMR Experiments

The NMR measurements were performed at Leibniz Institute for Analytical Sciences—ISAS, Dortmund, Germany. For ^1^H NMR profiling, 600 μL of deuterium oxide (D_2_O) (Sigma-Aldrich, Taufkirchen, Germany) was added to the lyophilized metabolite, in addition to an appropriate concentration of 3-(trimethylsilyl) propionate-2,2,3,3-d4 (TSP) as an internal reference and mixed thoroughly. Later, the samples were transferred into high resolution 5 mm borosilicate glass NMR tubes (Boro-600-5-8, Deutero GmbH, Kastellaun, Germany). The high resolution ^1^H NMR spectra of the intracellular extracted samples in addition to two reference samples were acquired using a broadband high resolution 600.13 MHz (B_0_ = 14.1 T) NMR Bruker spectrometer (Avance III 600, Bruker BioSpin GmbH, Rheinstetten, Germany) and a room-temperature NMR probe (BBO model-Bruker) at 279 K. Acquisition and processing of NMR spectra was achieved by using the Bruker TopSpin 3.6. The 1D NMR spectra were acquired using the 90° single-pulse experiment (Bruker pulse sequence zg) with embedded excitation sculpting for water suppression. ^1^H-^1^H TOCSY was acquired by employing the phase-sensitive TOCSY experiment, using *z*-axis decoupling in the presence of scalar interactions (DIPSI)-2 spin-lock implemented in the Bruker pulse sequence dipsi2esgpph. The spectral range was set to 7 kHz in both dimensions, 16K and 128 data points acquired in the horizontal and the vertical dimension (F2, F1), respectively. Before 2D Fourier Transform, zero filling was performed to 32K and 1K data points in the horizontal and the vertical dimension, respectively. The spectral widths in the two dimensions were 12.00 ppm.

### 2.4. Metabolic Profiling Assignment

**Figure 2:**
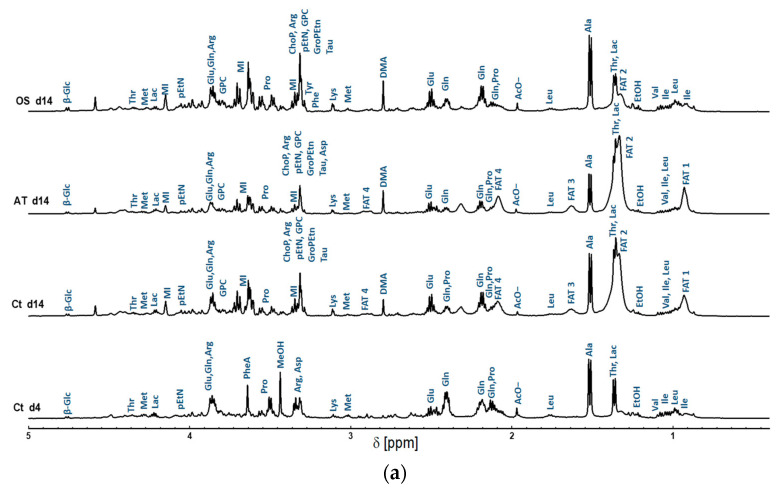

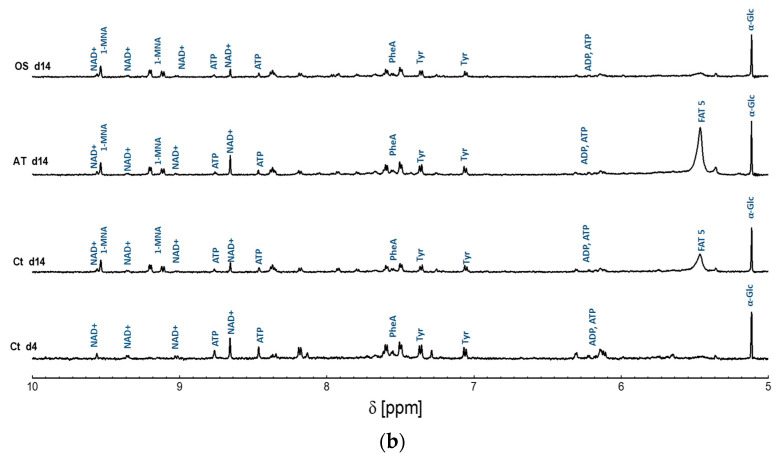
Representative high resolution ^1^H NMR spectra of intracellular metabolite extracts obtained from AT-derived hMSC samples collected at day 14 of differentiation into adipocytes and osteocytes, and their control samples represented in AT-derived hMSC collected at day 4 and 14 of cultivation in BCM. (**a**) 0.4–5 ppm region; (**b**) 5–10 ppm region. Peak assignment: Ile: Isoleucine; Leu: Leucine; Val: Valine; Thr: Threonine; Lac: Lactate; Ala: Alanine; Glu: Glutamine; Gln: Glutamate; Pro: Proline; Met: Methionine; Lys: Lysine; Arg: Arginine; GPC: Glycerophosphorylcholine; α-Glc: Alfa-Glucose; β-Glc: Beta-Glucose; MI: myo-inositol; ChoP: O-Phosphocholine; pEtN: Phosphorylethanolamine; GroPEtn: Glycerophosphorylethanolamine; ATP: Adenosine triphosphate; ADP: Adenosine diphosphate; Tyr: Tyrosine; Phe: Phenylalanine; NAD+: Nicotinamide adenine dinucleotide; Tau: Taurine; Asp: Asparagine; 1-MNA: 1-methylnicotinamide; AcO-: Acetate; DMA; Dimethylamine. In addition to the fatty acids signals; namely FAT 1, FAT 2, FAT 3, FAT 4, and FAT 5, representing methyl group -CH_3_, Acyl chains-(CH_2_)n-, methylene group -CH_2_-CH=CH, vinyl hydrogen -CH=CH, and diallyl methylene group =CH-CH_2_-CH=, respectively. The presence of ETOH (ethanol) and MeOH (methanol) was observed to represent residues from the cleaning and extraction procedures.

The metabolites were initially assigned using the high resolution 1D ^1^H NMR spectra of the studied MSCs in this work. One-dimensional ^1^H NMR high resolution spectra processing and pre-analysis were achieved using the TopSpin3.6, and metabolic assignment was accomplished using BMRB [22], HMAB [11] and Chenomx NMR Analysis Software. The detected metabolites were identified and annotated in the 1D spectra as shown in Figure 2.

The initial processing of the 2D NMR spectra was conducted using TopSpin 3.6 as follows: the spectra were referenced to the 2D contour of TSP and base levels were equalized to eliminate background noise. Later, automated peak picking at a proper threshold was performed by applying the automatic method using the pp2 function in TopSpin 3.6, and then the obtained F2 and F1 frequencies were determined.

**Figure 3:**
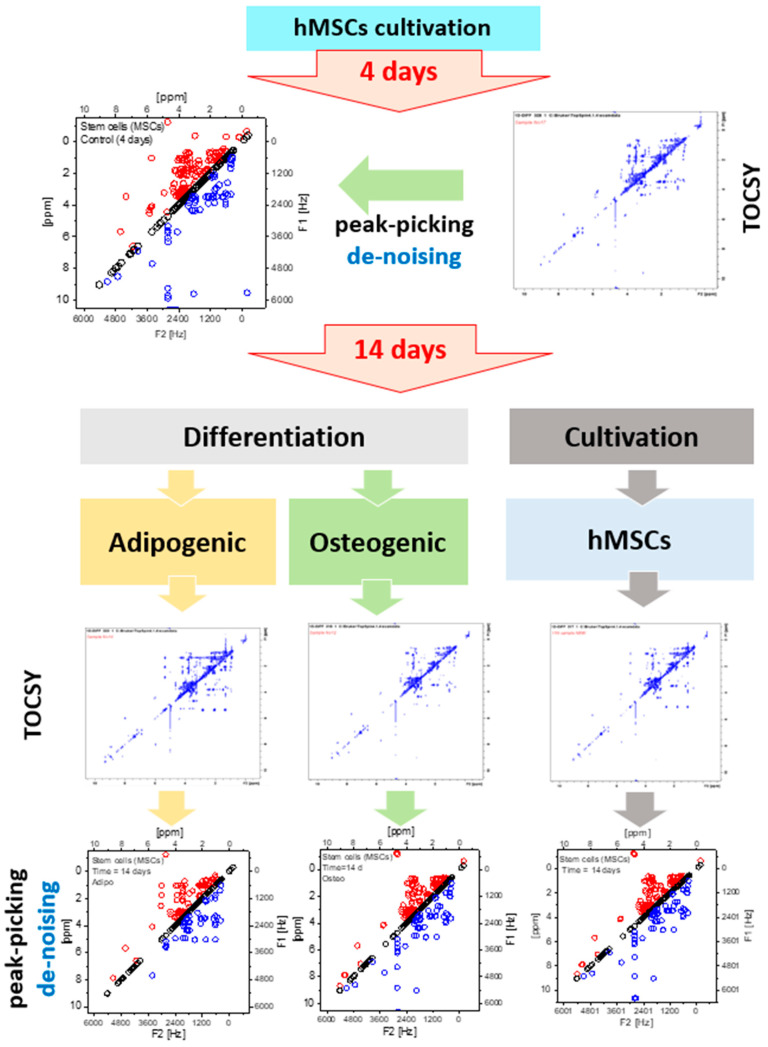
Schematic diagram of the experimental setting to observe the metabolic evolution of AT-derived hMSCs using 2D TOCSY of intracellular extracts of MSCs cultivated in basal culture media at 4 and 14 days and MSCs cultivated for a duration of 14 days in adipogenic and osteogenic differentiation media.

In agreement with the 1D spectra, a total of 32 metabolites were assigned from the 2D NMR spectra as shown in Table 1. Metabolites with only a single signal do not appear in the TOCSY spectrum. Taurine and asparagine were only detectable in the 2D spectra because they fully overlapped in the 1D spectra. Table 1 contains the metabolite name in the first column, and F2 and F1 measured the frequencies of the “Ct d4”, “Ct d14”, “AT d14” and “OS d14” samples. In the last column of Table 1, the standard F2 and F1 frequencies are listed. It can be observed that some metabolites appear and disappear during the cultivation and differentiation of the cells. In Table 1, the abbreviation ‘NP’ stands for ‘not present’ and it exposes the disappearance of metabolites during the dynamic evolution of the cells. Looking at the obtained metabolic 1D and 2D NMR spectra, metabolic changes occurring in the MSCs in response to prolonged cultivation or differentiation are noticeable and are mainly presented in their lipid profiles; this was shown by the different chemical groups corresponding to fatty acids. Multiple peaks, corresponding to the presence of chemical groups related to fatty acids that are normally produced by adipocytes, were predominant in the 1D and 2D NMR spectra of the differentiated and prolonged cultivation. MSCs differentiation is strongly related to remodeling in lipidomic metabolism directed by a variation in membrane demands depending on the differentiation characteristics and functional phenotypes [7,56,57,58]. Due to the variation of the level of intracellular metabolites, equalizing the signal intensities between all TOSCY NMR spectra leads to the disappearance of peaks with a signal to noise ratio (SNR) of less than three, as shown in Figure 2. A schematic diagram of the experimental results of this work is shown in Figure 3. AT-derived hMSCs are cultivated in a basal culture media and measured after four days using NMR. Non-targeted metabolic profiling of 2D NMR TOCSY is generated based on the four days’ cultivation where all collected peaks are manually assigned by the expert. AT-MSCs were subdivided into three experiments. In the first one, the MSCs were maintained in basal MSCs culture for prolonged cultivation. In the second and third experiments, AT-MSCs were induced to differentiate into adipocytes or osteocytes, respectively. After fourteen days, the adipogenic and osteogenic differentiation of the AT-derived hMSCs in addition to their control group were measured using 2D NMR TOCSY. Similarly, peak-picking was applied and the cross peaks were assigned by an expert. To evaluate the performance of our methodology, the manual assignments were compared to the automated method.

## 3. Datasets

In machine learning, creating a training model using diverse and large training dataset is crucial. Nevertheless, a reliable, large and labeled data set which considers the chemical shift and peak overlap does not exist. Using data augmentation [45,59], an extended data set of the peaks corresponding to the metabolites appearing in Table 1 is created. Multiple versions of the same metabolite are created by shifting the experimental chemical shift right and left up to 30 Hz to create the training dataset and adding random Gaussian noise to create the validation dataset [45,60]. Data augmentation is applied on the “control group at 4 days cultivation (Ct d4)” to create the training dataset.

The training dataset consists of 4000 independent data instances comprising all metabolites found on “Ct d4”. The horizontal and vertical frequencies of the TOCSY spectrum represent the features of the metabolites and the corresponding multiplet.

**Figure 4:**
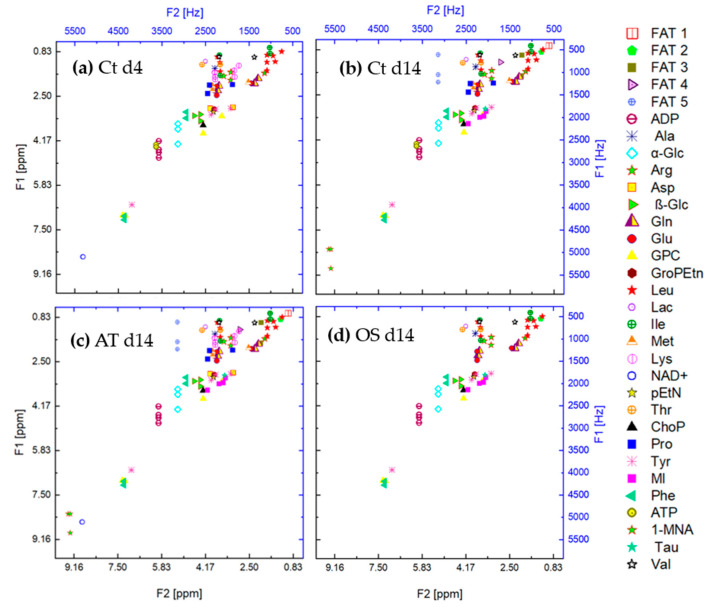
Feature space of the cross peaks of the metabolites contained in the samples (**a**) Ct d4, (**b**) Ct d14, (**c**) AT d14 and (**d**) OS d14.

Due to the different number of multiples per metabolite, an uneven distribution of classes in the training dataset is observed and a class imbalance problem can arise. To overcome this issue, under-sampling of metabolites with more than two multiples has been applied during the data augmentation procedure. Figure 4 shows the feature space of the metabolites contained in the cross peaks of the metabolites contained in the samples Ct d4, Ct d14 (control group at 14 days of cultivation), AT d14 (after 14 days of adipogenic differentiation) and OS d14 (after 14 days of osteogenic differentiation). It can be observed that the peaks overlap on the horizontal and vertical axes and cannot be linearly separated.

## 4. Results and Discussion of the Metabolic Evolution of AT-Derived hMSCs

To observe the dynamic of the AT-derived hMSCs at 14 days of cultivation (Ct d14), adipocytes (AT d14) and osteocytes (OS d14) after 14 days of differentiation, the training dataset created from (Ct d4) is used to create the main training model $\theta_{\mathrm{Ct}\;d4}$ using KNFST and KDE. Three independent testing datasets are constructed using Ct d14, AT d14 and OS d14 using the corresponding frequencies in Table 1, and are introduced to the classifiers and tested against $\theta_{\mathrm{Ct}\;d4}$.

**Figure 5:**
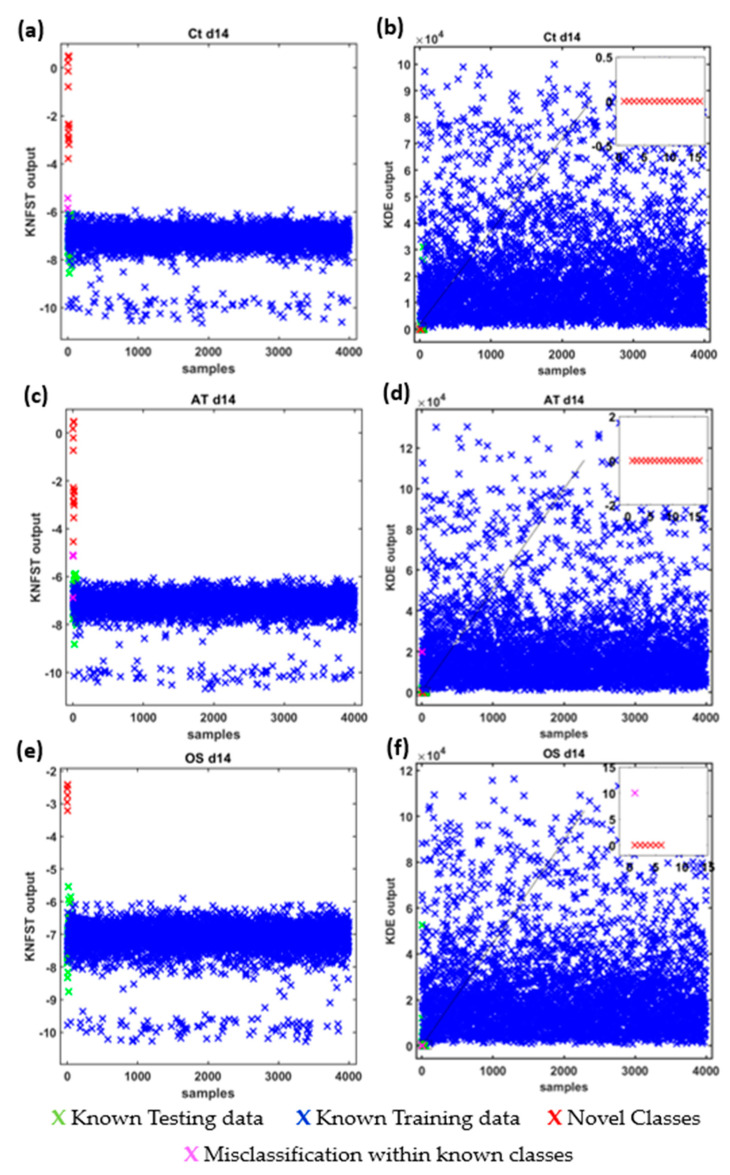
Novelty scores and threshold values of KDE and KNFST classifiers for Ct d14 (**a**,**b**), AT d14 (**c**,**d**) and OS d14 (**e**,**f**).

The results are reported as multi-class confusion matrices that compare the human-based metabolic profiling with the predicted assignments of the frequencies of the TOCSY spectra. In addition, Figure 5 shows the novelty scores produced by the classifiers are plotted to show the separation ability of the classifier in terms of projection distance for KNFST and probability estimation for KDE. The scores are color-coded to distinguish the scores of the different representations of classifier outputs as follows: the scores of known instances in the training set in blue, the scores of known instances in the testing dataset in green, the scores of missed novel instances in pink, the scores of correctly classified novel classes in red and the scores of misclassified known instances in the testing dataset in black. In ideal cases, the scores of known classes in the training dataset and testing dataset are similar. On the other hand, the scores of novel instances must be relatively different to those known classes. Novelty thresholds are created based on the validating dataset choosing the thresholds with a minimum validation error.

**Figure 6:**
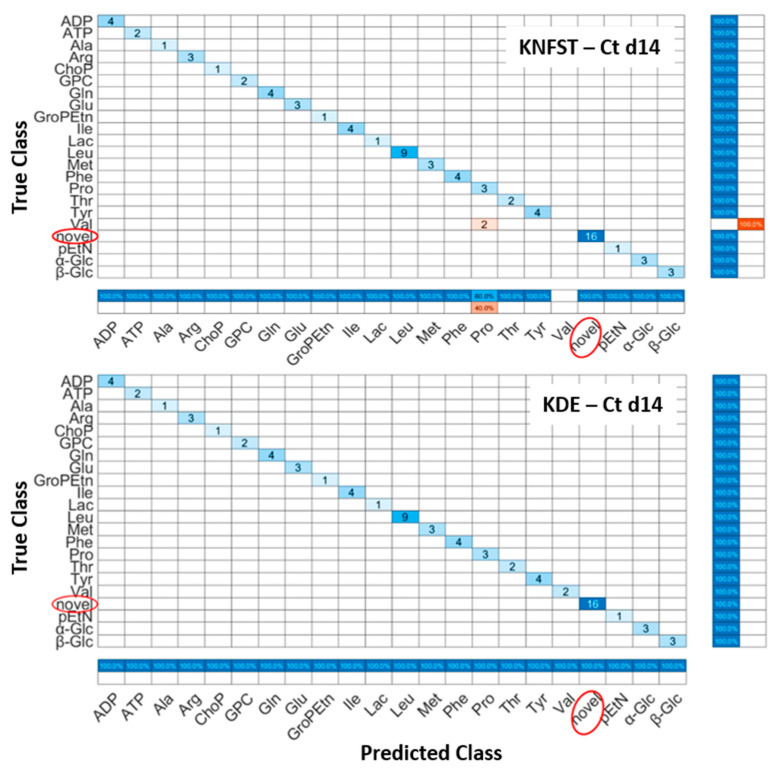
Confusion matrices of the output of classifiers KNFST and KDE for the spectra of 14 days of cultivation.

Ct d14: Figure 6 shows the confusion matrices for the output of the classifier KNFST and KDE for the Ct d14 sample. Both classifiers were able to detect all the sixteen novel frequencies which belong the fatty acids, 1-methylnicotinamide, myo-inositol, and taurine in the sample. No misclassification was encountered in KDE. This can be observed in Figure 5b, where the output of the known testing data, training data and novel classes are clearly distinct. Nevertheless, KNFST had two misclassifications within known classes, where the two instances of valine were misclassified as proline. This can be seen in Figure 5a, where two instances were plotted in pink, indicating the misclassification within known classes.

**Figure 7:**
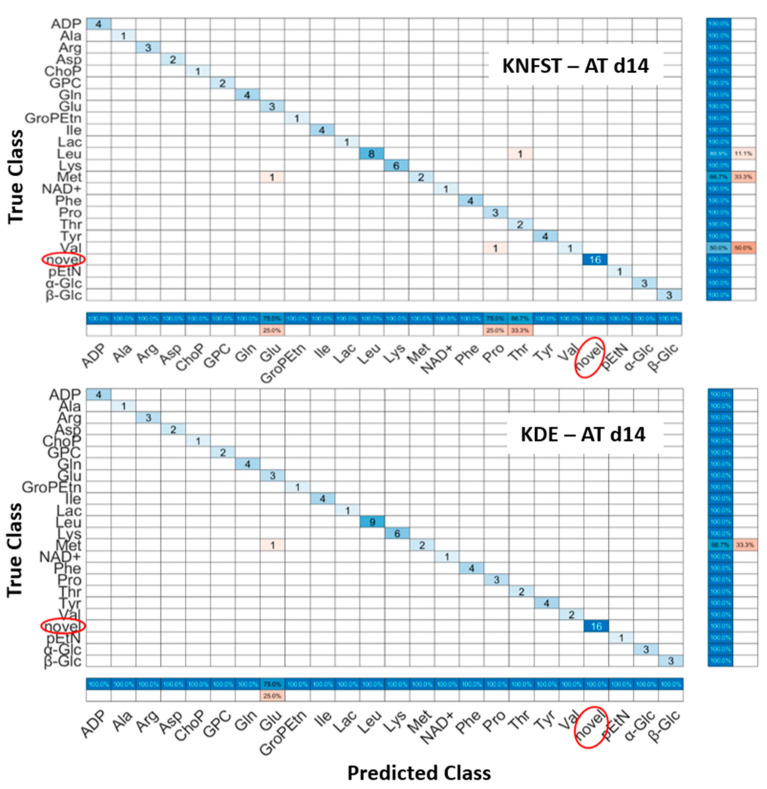
Confusion matrices of the output of classifiers KNFST and KDE for the spectra of 14 days of adipocyte differentiation.

AT d14: It can be seen in Figure 7, that both classifiers predicted all the sixteen novel metabolites which belong to the fatty acids, 1-methylnicotinamide, myo-inositol, and taurine in the sample. Nevertheless, both classifiers had misclassification within already known classes. KNFST and KDE misclassified methionine as glutamine. In addition, KNFST misclassified one of the instances of valine and proline as well as misclassified one instance of leucine as threonine. This can also be seen in Figure 5c,d, where misclassifications of known classes were plotted in pink.

**Figure 8:**
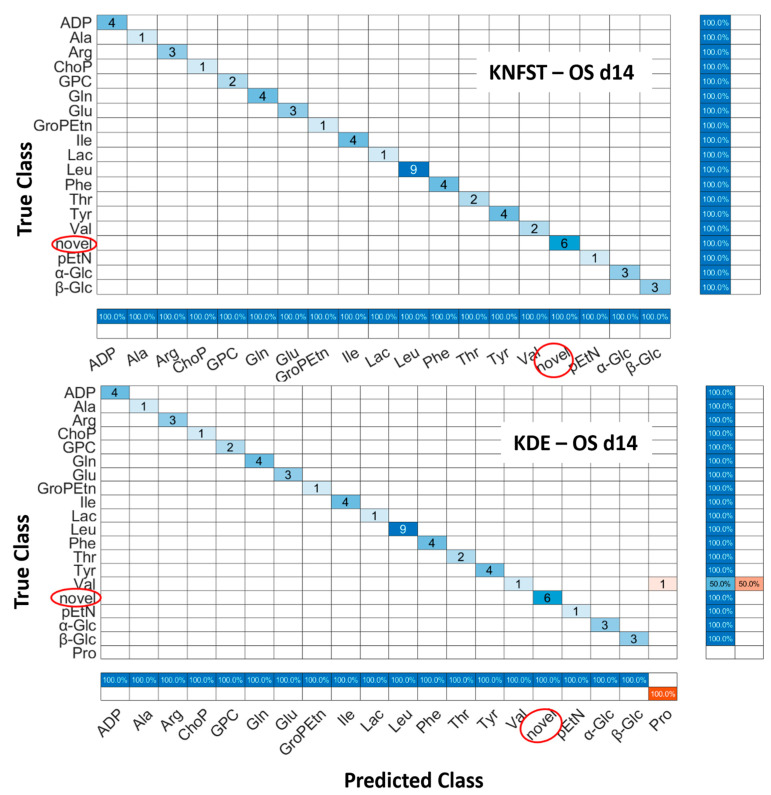
Confusion matrices of the output of classifiers KNFST and KDE for the spectra of 14 days of osteocyte differentiation.

OS d14: Figure 8 shows the confusion matrices for the output of the classifier KNFST and KDE for the OS d14 sample. Both classifiers were able to detect all six novel instances in the sample, such as myo-inositol, Fat2 and taurine. However, it can be observed that valine was misclassified as proline in KDE. This may be due to the overlap in the vertical and horizontal frequencies between these metabolites, which can be seen in Table 1 and Figure 4d. Except for this single misclassification, no misclassification was encountered in both classifiers. This can be also observed in Figure 5e,f.

Depending on the test sample, the number and type of novel metabolites differ. For instance, there are 16 identical novel (but shifted in frequency) metabolites in Ct d14 and AT d14 in comparison to Ct d4. Nevertheless, the disappearance of metabolites in both of these samples is also different. In sample OS d14, six metabolites were found in comparison to Ct d4, and more metabolites disappeared during the differentiation. For both classifiers and all samples, the disappearance of metabolites during the biological pathway did not affect the classification performance. For instance, though the main training model $\theta_{\mathrm{Ct}\;d4}$ was created on specific metabolites that disappeared in the spectra of Ct d14, AT d14 and OS d14, both classifiers proved their classification flexibility in observing metabolites presence and absence. Hence, the classifiers were able to detect both the presence and the absence of individual metabolites in accordance with $\theta_{\mathrm{Ct}\;d4}$.

Following the novelty detection metrics used in [61], the assessment measures are:

(1) False negative rate =(100*Fn)/(Nn)False positive rate =(100*Fp)/(N−Nn)Total error=(100*Error)/(N)

where Fn is the number of novel metabolites classified as known, Nn is the number of novel instances in the test dataset, N is the total number of instances in the test dataset, Fp is the number of known metabolites misclassified as novel metabolites and Fe is the misclassifications within known metabolites and Error=Fn+Fe+Fp. Table 2 shows the results following these assessment measures. It can be seen that no false positive or false negative error was encountered. However, the most recurrent error was related to the misclassification within known classes which can be associated with the overlap in the frequency between these metabolites.

## 5. Conclusions

This article demonstrates using machine learning to perform an automatic analysis of ^1^H-^1^H TOCSY spectra acquired on cultivated and differentiated adipose-tissue-derived human MSCs (AT-derived hMSCs). Multi-class classification in addition to the novelty detection of metabolites were established based on four different 2D NMR TOCSY spectra. The primary training model was built using TOCSY spectrum of AT-derived hMSCs at four days of cultivation. Subsequently, the metabolic changes of AT-derived hMSCs control sample was monitored under three different biological settings employing the classifiers KDE and KNFST. In spite of the severe overlapping in the frequencies in TOCSY spectra, the classification outputs proved the efficiency of the used method. KDE and KNFST achieved a total classification error between 0% and 3.6% and false positive and false negative rates of 0%. The investigation in this work confirms the common metabolic pathways associated to stem cell biology. In the future, further features can be added to the dataset to produce a higher discriminative ability. Furthermore, chemical structure information or integrating other 2D NMR spectra can be included in the classification process. This work provides methodological approaches to track information of MSCs metabolism and their biological pathways, including detecting novel metabolites related to diverse stimuli in terms of prolonged cultivation and varied differentiation. This work can be extended to monitor further kinds of MSCs proliferation and recognize spectral signatures of pathways and processes.

## Figures and Tables

**Table 1:** Intracellular metabolites detected in AT-derived hMSCs. Frequencies: control group at 4 days of cultivation (Ct d4), 14 days of cultivation (Ct d14), 14 days of differentiation into adipocytes (AT d14) and osteocytes (OS d14). NP: Not present.

Metabolite	Ct d4		Ct d14		AT d14		OS d14		Standard	
	F2	F1	F2	F1	F2	F1	F2	F1	F2	F1
Leu	752	494	762	484	762	489	772	494	720	540
Leu	950	590	930	584	930	589	934	560	900	600
Leu	900	720	914	720	913	750	910	760	900	720
Leu	1040	610	1073	631	1060	638	1046	608	1080	600
Leu	1040	695	1073	705	1060	709	1046	749	1080	720
Leu	1040	893	1073	923	1060	912	1046	922	1080	900
Leu	2229	611	2210	608	2212	610	2198	620	2220	600
Leu	2170	737	2171	750	2171	752	2163	749	2220	720
Leu	2163	943	2168	943	2157	908	2175	921	2220	900
Ile	1020	540	1020	540	1020	540	1020	540	1020	540
Ile	1023	408	1009	403	1047	417	1045	417	1020	600
Ile	2180	570	2170	580	2178	578	2220	540	2220	540
Ile	2160	1032	2165	1042	2195	1052	2210	1003	2220	1020
Tyr	1920	1778	1920	1787	1920	1782	1920	1790	1920	1830
Tyr	2396	1788	2253	1784	2355	1786	2358	1780	2340	1830
Tyr	2304	1877	2253	1848	2356	1836	2356	1848	2362	1920
Tyr	4073	4067	4090	3946	4094	3961	4095	3952	4316	4139
Phe	2340	1853	2354	1906	2261	1778	2360	1848	2390	1868
Phe	2340	1934	2254	1960	2254	1926	2260	1913	2390	1970
Phe	4362	4193	4362	4193	4368	4193	4370	4197	4453	4422
Phe	4362	4273	4362	4275	4368	4273	4370	4286	4453	4394
Glu	1373	1102	1354	1106	1354	1106	1349	1106	1470	1260
Glu	2337	1043	2337	1057	2344	1048	2333	1062	2258	1278
Glu	2341	1278	2344	1269	2341	1288	2333	1278	2258	1468
Gln	1295	1100	1281	1100	1284	1071	1288	1100	1260	1200
Gln	1378	1220	1384	1200	1389	1210	1370	1230	1380	1200
Gln	2190	1269	2186	1288	2191	1288	2194	1288	2220	1200
Gln	2225	1370	2227	1367	2210	1380	2208	1369	2220	1380
Lys	1740	809	NP	NP	1736	783	NP	NP	1800	840
Lys	1844	893	NP	NP	1836	898	NP	NP	1800	900
Lys	1836	1062	NP	NP	1836	1058	NP	NP	1806	1032
Lys	2295	962	NP	NP	2290	962	NP	NP	2220	900
Lys	2282	1057	NP	NP	2278	1044	NP	NP	2250	1032
Lys	2282	1118	NP	NP	2286	1119	NP	NP	2250	1137
FAT 1	NP	NP	616	405	600	420	NP	NP	600	420
FAT 2	NP	NP	789	545	789	531	789	545	785	535
FAT 3	NP	NP	1230	614	1245	620	NP	NP	1260	600
FAT 3	NP	NP	1240	1080	1260	1080	NP	NP	1260	1050
FAT 4	NP	NP	1715	772	1705	778	NP	NP	1792	766
FAT 5	NP	NP	3139	607	3150	607	NP	NP	3180	540
FAT 5	NP	NP	3138	1052	3150	1052	NP	NP	3180	1080
FAT 5	NP	NP	3140	1217	3150	1219	NP	NP	3180	1260
Lac	2499	715	2494	709	2494	715	2494	720	2463	790
Thr	2160	789	2160	790	2160	720	2160	720	2160	780
Thr	2578	789	2537	790	2582	720	2573	720	2580	780
Pro	1879	1238	1869	1230	1873	1238	NP	NP	1980	1200
Pro	2408	1246	2408	1234	2408	1238	NP	NP	2472	1213
Pro	2408	1438	2435	1448	2405	1439	NP	NP	2472	1402
Ala	2270	723	2295	696	2295	705	2291	701	2256	876
Val	1350	632	1383	619	1394	619	1383	619	1380	617
Val	1875	1237	1890	1259	1880	1244	1870	1240	2160	617
Met	1518	1187	1523	1197	1518	1177	NP	NP	1560	1260
Met	2338	1270	2342	1274	2351	1277	NP	NP	2340	1260
Met	2338	1370	2342	1367	2351	1380	NP	NP	2340	1320
pEtN	2317	1852	2305	1848	2331	1865	2307	1849	2430	1950
GroPEtn	2300	1791	2291	1781	2293	1791	2292	1791	2300	1791
ChoP	2454	1950	2455	1953	2458	1954	2454	1947	2572	2187
GPC	2127	1943	2121	1943	2124	1939	2123	1939	2160	1980
GPC	2552	2333	2535	2338	2544	2359	2533	2348	2580	2340
Arg	1144	1000	1140	975	1143	984	1146	980	1120	920
Arg	1910	960	1920	960	1944	988	1928	988	1920	960
Arg	1974	1134	1978	1134	1986	1115	1969	1130	1920	1140
MI	NP	NP	2039	1880	2044	1869	2036	1869	2040	1800
MI	NP	NP	2088	1882	2093	1872	2087	1866	2112	1959
MI	NP	NP	2154	1970	2159	1977	2152	1972	2167	1959
MI	NP	NP	2460	2156	2452	2140	2452	2149	2423	2113
Asp	2390	1574	NP	NP	2398	1578	NP	NP	2400	1800
Asp	1870	1750	NP	NP	1876	1768	NP	NP	1800	1740
Tau	NP	NP	2064	1809	2065	1812	2063	1812	2040	1980
α-Glc	3135	2119	3125	2139	3137	2132	3140	2112	3130	2112
α-Glc	3135	2238	3125	2254	3137	2263	3140	2280	3130	2224
α-Glc	3135	2573	3125	2558	3132	2562	3140	2565	3130	2568
β-Glc	2760	1937	2774	1928	2765	1931	2759	1936	2778	1938
β-Glc	2717	2055	2714	2065	2717	2063	2717	2068	2778	2084
β-Glc	2717	2008	2714	2000	2712	2002	2714	2089	2778	2081
ATP	3620	2587	3640	2581	NP	NP	NP	NP	3620	2587
ATP	3620	2680	3640	2628	NP	NP	NP	NP	3620	2680
ADP	3569	2496	3566	2503	3566	2501	3570	2498	3569	2496
ADP	3569	2700	3566	2706	3569	2708	3569	2690	3569	2700
ADP	3569	2762	3566	2759	3569	2769	3569	2765	3569	2760
ADP	3569	2882	3566	2885	3569	2870	3569	2868	3569	2880
NAD+	5310	5110	NP	NP	5302	5106	NP	NP	5200	5110
1-MNA	NP	NP	5218	4718	5218	4725	NP	NP	5341	4921
1-MNA	NP	NP	5412	4718	5412	4725	NP	NP	5581	4921
1-MNA	NP	NP	5520	5328	5512	5321	NP	NP	5581	5341

**Table 2:** A summary of the performance of KDE and KNFST classifiers for Ct d14, AT d14 and OS d14.

	Ct d14		AT d14		OS d14	
	KNFST	KDE	KNFST	KDE	KNFST	KDE
False negative rate	0%	0%	0%	0%	0%	0%
False positive rate	0%	0%	0%	0%	0%	0%
Total error	2.6%	0%	3.6%	1.2%	0%	1.7%

## Data Availability

Data available in a publicly accessible repository. The data presented in this study are openly available on Zenodo at: https://doi.org/10.5281/zenodo.7276518. (Accessed on 24 February 2023).

## References

[1] Saeedi P., Halabian R., Fooladi A.A.I. (2019). A revealing review of mesenchymal stem cells therapy, clinical perspectives and Modification strategies. Stem Cell Investig..

[2] Andrzejewska A., Lukomska B., Janowski M. (2019). Concise review: Mesenchymal stem cells: From roots to boost. Stem Cells.

[3] Alhattab D., Jamali F., Ali D., Hammad H., Adwan S., Rahmeh R., Samarah O., Salah B., Hamdan M., Awidi A. (2019). An insight into the whole transcriptome profile of four tissue-specific human mesenchymal stem cells. Regen. Med..

[4] Chu D.-T., Nguyen Thi Phuong T., Tien N.L.B., Tran D.K., Minh L.B., Van Thanh V., Gia Anh P., Pham V.H., Thi Nga V. (2019). Adipose Tissue Stem Cells for Therapy: An Update on the Progress of Isolation, Culture, Storage, and Clinical Application. J. Clin. Med..

[5] Funes J.M., Quintero M., Henderson S., Martinez D., Qureshi U., Westwood C., Clements M.O., Bourboulia D., Pedley R.B., Moncada S. (2007). Transformation of human mesenchymal stem cells increases their dependency on oxidative phosphorylation for energy production. Proc. Natl. Acad. Sci. USA.

[6] Rocha B., Calamia V., Mateos J., Fernández-Puente P., Blanco F.J., Ruiz-Romero C. (2012). Metabolic Labeling of Human Bone Marrow Mesenchymal Stem Cells for the Quantitative Analysis of their Chondrogenic Differentiation. J. Proteome Res..

[7] Bispo D.S.C., Jesus C.S.H., Correia M., Ferreira F., Bonifazio G., Goodfellow B.J., Oliveira M.B., Mano J.F., Gil A.M. (2022). NMR Metabolomics Assessment of Osteogenic Differentiation of Adipose-Tissue-Derived Mesenchymal Stem Cells. J. Proteome Res..

[8] Salazar-Noratto G.E., Luo G., Denoeud C., Padrona M., Moya A., Bensidhoum M., Bizios R., Potier E., Logeart-Avramoglou D., Petite H. (2019). Understanding and leveraging cell metabolism to enhance mesenchymal stem cell transplantation survival in tissue engineering and regenerative medicine applications. Stem Cells.

[9] Zhu H., Sun A., Zou Y., Ge J. (2014). Inducible Metabolic Adaptation Promotes Mesenchymal Stem Cell Therapy for Ischemia. Arter. Thromb. Vasc. Biol..

[10] Emwas A.-H., Roy R., McKay R.T., Tenori L., Saccenti E., Gowda G.A.N., Raftery D., Alahmari F., Jaremko L., Jaremko M. (2019). NMR Spectroscopy for Metabolomics Research. Metabolites.

[11] Wishart D.S., Jewison T., Guo A.C., Wilson M., Knox C., Liu Y., Djoumbou Y., Mandal R., Aziat F., Dong E. (2013). HMDB 3.0—The Human Metabolome Database in 2013. Nucleic Acids Res..

[12] Xu Z.-F., Pan A.-Z., Yong F., Shen C.-Y., Chen Y.-W., Wu R.-H. (2012). Human umbilical mesenchymal stem cell and its adipogenic differentiation: Profiling by nuclear magnetic resonance spectroscopy. World J. Stem Cells.

[13] Gowda G.A.N., Zhang S., Gu H., Asiago V., Shanaiah N., Raftery D. (2008). Metabolomics-based methods for early disease diagnostics. Expert Rev. Mol. Diagn..

[14] Bingol K., Zhang F., Bruschweiler-Li L., Brüschweiler R. (2013). Quantitative Analysis of Metabolic Mixtures by Two-Dimensional ^13^C Constant-Time TOCSY NMR Spectroscopy. Anal. Chem..

[15] Hao J., Liebeke M., Astle W., De Iorio M., Bundy J.G., Ebbels T. (2014). Bayesian deconvolution and quantification of metabolites in complex 1D NMR spectra using BATMAN. Nat. Protoc..

[16] Dona A.C., Kyriakides M., Scott F., Shephard E.A., Varshavi D., Veselkov K., Everett J.R. (2016). A guide to the identification of metabolites in NMR-based metabonomics/metabolomics experiments. Comput. Struct. Biotechnol. J..

[17] Güntert P. (2008). Automated structure determination from NMR spectra. Eur. Biophys. J..

[18] Ross A., Schlotterbeck G., Dieterle F., Senn H., Lindon J., Nicholson J., Holmes E. (2007). Chapter 3—NMR Spectroscopy Techniques for Application to Metabonomics. The Handbook of Metabonomics and Metabolomics.

[19] Roberts L.D., Souza A.L., Gerszten R.E., Clish C.B. (2012). Targeted Metabolomics. Curr. Protoc. Mol. Biol..

[20] Ai Z., Zhang Y., Li X., Sun W., Liu Y. (2021). Widely Targeted Metabolomics Analysis to Reveal Transformation Mechanism of Cistanche Deserticola Active Compounds During Steaming and Drying Processes. Front. Nutr..

[21] Bingol K., Li D.-W., Zhang B., Brüschweiler R. (2016). Comprehensive Metabolite Identification Strategy Using Multiple Two-Dimensional NMR Spectra of a Complex Mixture Implemented in the COLMARm Web Server. Anal. Chem..

[22] Ulrich E.L., Akutsu H., Doreleijers J.F., Harano Y., Ioannidis Y.E., Lin J., Livny M., Mading S., Maziuk D., Miller Z. (2007). BioMagResBank. Nucleic Acids Res..

[23] Cheng Y., Gao X., Liang F. (2014). Bayesian Peak Picking for NMR Spectra. Genom. Proteom. Bioinform..

[24] Klukowski P., Walczak M.J., Gonczarek A., Boudet J., Wider G. (2015). Computer vision-based automated peak picking applied to protein NMR spectra. Bioinformatics.

[25] Chen D., Wang Z., Guo D., Orekhov V., Qu X. (2020). Review and Prospect: Deep Learning in Nuclear Magnetic Resonance Spectroscopy. Chem. A Eur. J..

[26] Abadi M., Agarwal A., Barham P., Brevdo E., Chen Z., Citro C., Corrado G.S., Davis A., Dean J., Devin M. (2016). Tensorflow: Large-scale machine learning on heterogeneous distributed systems. 12th USENIX Symposium on Operating Systems Design and Implementation (OSDI 16).

[27] Hansen D.F. (2019). Using Deep Neural Networks to Reconstruct Non-uniformly Sampled NMR Spectra. J. Biomol. NMR.

[28] Kwon Y., Lee D., Choi Y.-S., Kang M., Kang S. (2020). Neural Message Passing for NMR Chemical Shift Prediction. J. Chem. Inf. Model..

[29] Lee H.H., Kim H. (2019). Intact metabolite spectrum mining by deep learning in proton magnetic resonance spectroscopy of the brain. Magn. Reson. Med..

[30] Zhang C., Idelbayev Y., Roberts N., Tao Y., Nannapaneni Y., Duggan B.M., Min J., Lin E.C., Gerwick E.C., Cottrell G.W. (2017). Small Molecule Accurate Recognition Technology (SMART) to Enhance Natural Products Research. Sci. Rep..

[31] Reher R., Kim H.W., Zhang C., Mao H.H., Wang M., Nothias L.-F., Caraballo-Rodriguez A.M., Glukhov E., Teke B., Leao T. (2020). A Convolutional Neural Network-Based Approach for the Rapid Annotation of Molecularly Diverse Natural Products. J. Am. Chem. Soc..

[32] Chopra S., Hadsell R., LeCun Y. Learning a similarity metric discriminatively, with application to face verification. Proceedings of the 2005 IEEE Computer Society Conference on Computer Vision and Pattern Recognition (CVPR’05).

[33] Klukowski P., Augoff M., Zięba M., Drwal M., Gonczarek A., Walczak M.J. (2018). NMRNet: A deep learning approach to automated peak picking of protein NMR spectra. Bioinformatics.

[34] Jang M.-Y., Chun S.-I., Mun C.-W., Hong K.S., Shin J.-W. (2013). Evaluation of Metabolomic Changes as a Biomarker of Chondrogenic Differentiation in 3D-cultured Human Mesenchymal Stem Cells Using Proton (1H) Nuclear Magnetic Resonance Spectroscopy. PLoS ONE.

[35] Bispo D.S.C., Michálková L., Correia M., Jesus C.S.H., Duarte I.F., Goodfellow B.J., Oliveira M.B., Mano J.F., Gil A.M. (2022). Endo- and Exometabolome Crosstalk in Mesenchymal Stem Cells Undergoing Osteogenic Differentiation. Cells.

[36] Castiglione F., Ferro M., Mavroudakis E., Pellitteri R., Bossolasco P., Zaccheo D., Morbidelli M., Silani V., Mele A., Moscatelli D. (2017). NMR Metabolomics for Stem Cell type discrimination. Sci. Rep..

[37] Coope A., Ghanameh Z., Kingston O., Sheridan C.M., Barrett-Jolley R., Phelan M.M., Oldershaw R.A. (2022). ^1^H NMR Metabolite Monitoring during the Differentiation of Human Induced Pluripotent Stem Cells Provides New Insights into the Molecular Events That Regulate Embryonic Chondrogenesis. Int. J. Mol. Sci..

[38] Roberts S.J. Extreme value statistics for novelty detection in biomedical signal processing. Proceedings of the 2000 First International Conference Advances in Medical Signal and Information Processing.

[39] Markou M., Singh S. (2003). Novelty detection: A review—part 1: Statistical approaches. Signal Process..

[40] Pimentel M.A., Clifton D.A., Clifton L., Tarassenko L. (2014). A review of novelty detection. Signal Process..

[41] Bishop C. (1994). Novelty detection and neural network validation. IEE Proc.—Vision, Image, Signal Process..

[42] Bodesheim P., Freytag A., Rodner E., Kemmler M., Denzler J. Kernel Null Space Methods for Novelty Detection. Proceedings of the IEEE Computer Society Conference on Computer Vision and Pattern Recognition.

[43] Huang X., Xu J., Guo G. Incremental Kernel Null Foley-Sammon Transform for Person Re-identification. Proceedings of the 2018 24th International Conference on Pattern Recognition (ICPR).

[44] Gu G., Liu H., Shen J. Kernel null foley-sammon transform. Proceedings of the 2008 International Conference on Computer Science and Software Engineering.

[45] Migdadi L., Lambert J., Telfah A., Hergenröder R., Wöhler C. (2021). Automated metabolic assignment: Semi-supervised learning in metabolic analysis employing two dimensional Nuclear Magnetic Resonance (NMR). Comput. Struct. Biotechnol. J..

[46] Parzen E. (1962). On Estimation of a Probability Density Function and Mode. Ann. Math. Stat..

[47] Bishop C.M. (2006). Pattern Recognition and Machine Learning 2006.

[48] Migdadi L., Telfah A., Hergenröder R., Wöhler C. (2022). Novelty detection for metabolic dynamics established on breast cancer tissue using 2D NMR TOCSY spectra. Comput. Struct. Biotechnol. J..

[49] Abuarqoub D., Awidi A., Abuharfeil N. (2015). Comparison of osteo/odontogenic differentiation of human adult dental pulp stem cells and stem cells from apical papilla in the presence of platelet lysate. Arch. Oral Biol..

[50] Yoon J.-Y. (2022). Cell Culture. Tissue Engineering: A Primer with Laboratory Demonstrations.

[51] Ghorbani A., Jalali S.A., Varedi M. (2014). Isolation of adipose tissue mesenchymal stem cells without tissue destruction: A non-enzymatic method. Tissue Cell.

[52] Sathishkumar S., Mohanashankar P., Boopalan P. (2011). Cell surface protein expression of stem cells from human adipose tissue at early passage with reference to mesenchymal stem cell phenotype. Int. J. Med. Med. Sci..

[53] Zhang A.-X., Yu W.-H., Ma B.-F., Yu X.-B., Mao F.F., Liu W., Zhang J.-Q., Zhang X.-M., Li S.-N., Li M.-T. (2007). Proteomic identification of differently expressed proteins responsible for osteoblast differentiation from human mesenchymal stem cells. Mol. Cell. Biochem..

[54] Umrath F., Weber M., Reinert S., Wendel H.-P., Avci-Adali M., Alexander D. (2020). iPSC-Derived MSCs versus Originating Jaw Periosteal Cells: Comparison of Resulting Phenotype and Stem Cell Potential. Int. J. Mol. Sci..

[55] Martineau E., Tea I., Loaëc G., Giraudeau P., Akoka S. (2011). Strategy for choosing extraction procedures for NMR-based metabolomic analysis of mammalian cells. Anal. Bioanal. Chem..

[56] Levental K.R., Surma M.A., Skinkle A.D., Lorent J.H., Zhou Y., Klose C., Chang J.T., Hancock J.F., Levental I. (2017). ω-3 polyunsaturated fatty acids direct differentiation of the membrane phenotype in mesenchymal stem cells to potentiate osteogenesis. Sci. Adv..

[57] Assis-Ribas T., Forni M.F., Winnischofer S.M.B., Sogayar M., Trombetta-Lima M. (2018). Extracellular matrix dynamics during mesenchymal stem cells differentiation. Dev. Biol..

[58] Shi C., Wang X., Wu S., Zhu Y., Chung L.W.K., Mao H. (2008). HRMAS^1^H-NMR measured changes of the metabolite profile as mesenchymal stem cells differentiate to targeted fat cells in vitro: Implications for non-invasive monitoring of stem cell differentiation in vivo. J. Tissue Eng. Regen. Med..

[59] Wong S.C., Gatt A., Stamatescu V., McDonnell M.D. Understanding Data Augmentation for Classification: When to Warp?. Proceedings of the 2016 International Conference on Digital Image Computing: Techniques and Applications (DICTA).

[60] Liu J., Osadchy M., Ashton L., Foster M., Solomon C.J., Gibson S.J. (2017). Deep convolutional neural networks for Raman spectrum recognition: A unified solution. Analyst.

[61] Masud M., Gao J., Khan L., Han J., Thuraisingham B.M. (2010). Classification and Novel Class Detection in Concept-Drifting Data Streams under Time Constraints. IEEE Trans. Knowl. Data Eng..

